# Influence of the sanitary sewage application method to closed-end furrows on the macronutrient extraction capacity and productivity of elephant grass

**DOI:** 10.1038/s41598-020-77038-6

**Published:** 2020-11-17

**Authors:** Marcus Vinícius Araújo Marques, Antonio Teixeira de Matos, Thiago Henrique Ribeiro Silvério, Ana Paula Miranda Pereira

**Affiliations:** Department of Sanitary and Environmental Engineering, Federal University of Minas Gerais (Universidade Federal de Minas Gerais), Belo Horizonte, Minas Gerais Brazil

**Keywords:** Environmental monitoring, Element cycles, Environmental impact, Plant evolution, Leaf development

## Abstract

The objective of this work was to evaluate the influence of the form of raw sanitary sewage (RS) application in closed-end and level-bottom furrows on the dry matter yield and macronutrient extraction capacity (extraction of nitrogen, phosphorus and potassium) by the aerial part (stem and leaves) of elephant grass. Fertigation of elephant grass with RS were conducted for 3 uninterrupted years, and the RS application dose was established as a function of the amount of sodium fed to the soil (300 kg ha^−1^ year^−1^). In the experimental planning four treatments were established, where two experimental plots received RS and the water demand was complemented by treated water from the public supply network (TW), with and without alternation in the position of RS application in the furrows (TFA and TFN, respectively); and as a control there were two experimental plots in which the plants received conventional mineral fertilization, where the grass was irrigated with TW, with and without alternation in the position of water application in the furrows (TWA and TWN, respectively). The greatest mean dry matter yield (29.9 Mg ha^−1^ year^−1^) and mean macronutrient extraction values were found for plants submitted to TFA (688, 102, 508 kg ha^−1^ year^−1^ of N, P and K, respectively), compared to those obtained in the other treatments.

## Introduction

The use of raw sanitary sewage (RS) in fertigation is a source of nutrients for plants, allowing for reduced application of mineral fertilizers to agricultural crops^1^, as well as supplying part of the water needs of plants. Agricultural use of RS is based on the certainty of its availability throughout the year, especially in cities with a sewage system network, in addition to reducing the costs of its treatment for release into water bodies^2^.

Given these possibilities, application engineering and systems management are subjects that require further technological development. The most commonly used application techniques are spraying and dripping. Irrigation by furrows, although considered a rudimentary form of agricultural crop irrigation, can currently be considered among the most appropriate when dealing with raw wastewater because it presents a low risk of blocking emitters by chemical, physical and biological agents compared to local applications^3–5^. This method also presents better sanitation safety, without contamination of plants and system operators compared to application by spraying^6^.

However, furrow fertigation technology presents problems with respect to soil nutrient distribution, making system management difficult. This has required the dedication of several researchers in proposing recommendations to optimize this process^7–11^. Studies have shown that the application of nutrients via conventional fertigation by furrows, i.e., maintaining the same application position in the furrows, can cause the accumulation of nutrients and pollutants near the application site which is at the inlet of the furrows^12,13^.

An important point in sizing and observing the safety of the fertigation process with RS is the ability of the plant aerial part to extract nutrients, because this factor indicates how much the crop is contributing to the removal of nutrients and sodium applied to the soil via RS^14^.

The macronutrient extraction capacity of elephant grass (an important forage species in Brazilian livestock) depends mainly on soil fertilization and crop management^15,16^. The influence of different fertilizer doses, types of fertilizers applied, cutting ages, season, cultivars and other variables that affect productivity of this grass has been addressed in several studies^17–19^, however the response of this plant to application of sanitary sewage has not been mentioned.

The objective of the present work was to evaluate the influence of the RS application position in flat bottom furrows with closed ends on the productivity and macronutrient extraction capacity (extraction of nitrogen, phosphorus and potassium) of elephant grass, comparing the results with those obtained with conventional mineral fertilization (CMF), during 3 years of uninterrupted experimentation.

## Materials and methods

The experiment was conducted in an area at the Sewage Treatment Station of the Minas Gerais Sanitation Company (*Companhia de Saneamento de Minas Gerais—COPASA ETE—Onça*), located near Ribeirão do Onça, in Santa Luzia—MG. This is near Belo Horizonte—MG, Brazil, at the geographic coordinates 19°49′20.6″ South and 43°53′46.6″ West, at an elevation of 852 m.

The region has a humid tropical climate, with most rainfall during the summer. The soil in the area was formed from compacted material, and it was therefore not possible to obtain proper classification, although according to the Soil Taxonomy of the United States Department of Agriculture^20^, it has similar characteristics to an Inceptisol.

Elephant grass (*Pennisetum purpureum*) was planted in June 2016, from plant stalks. The experiment was setup in a completely randomized design with 4 treatments and 7 sampling regions along the length of the furrows, with 7 replicates each treatment. The treatments considered were: (i) conventional mineral fertilization (CMF) and irrigation with treated water (TW) applied without alternation in the position of water application in the furrows (TWN); (ii) CMF and irrigation with TW applied with alternation in furrow application position (TWA); (iii) Fertigation with raw sanitary sewage (RS) and complemented with TW irrigation, both applied without alternation in the furrow application position (TFN); and (iv) Fertigation with RS and complemented with TW irrigation, both applied with alternation in the furrow application position (TFA). Each experimental plot was 72 m^2^ (total of 288 m^2^ for each 4 treatments), which consisted of three furrows and four planted rows, where the lateral furrows and lateral planted rows were disregarded in the analyzes since they were used only as “borders” to reduce external interference with the system under analysis. The length of the furrows was 40 m, with a spacing of 0.6 m between them, built on level ground and with closed ends.

In the experimental plots that received conventional mineral fertilization (CMF), the doses of 70 kg ha^−1^ year^−1^ of P were applied manually at the beginning of each year, and 300 kg ha^−1^ year^−1^ of N and K were applied manually in equal doses after each cutting of the aerial part of the plants, using the commercial fertilizers simple superphosphate, urea and potassium chloride, according to the recommendations of the Soil Fertility Commission of Minas Gerais State^21^.

Fertigation with RS was performed weekly by applying a dosage equivalent to 300 kg ha^−1^ year^−1^ of Na^22^. Irrigation and complementing water application to the plots fertigated with RS were also performed weekly, and evapotranspiration was used to calculate the water demands of the plants^23^. The monthly and annual average temperature (T_mean_), monthly and annual average relative humidity (RH_mean_), and monthly and annual accumulated average precipitation (Pr) during the experimental period are presented in Table 1^24^.

**Table 1:** Average monthly and annual temperature (T_mean_), relative humidity (RH_mean_), and accumulated precipitation (Pr) during the experiment period. Source: INMET—Instituto Nacional de Meteorologia^24^.

Month	T_mean_ (ºC)	RH_mean_ (%)	Pr (mm)
January	25	58	133
February	24	68	193
March	24	65	211
April	23	65	50
May	21	64	28
June	20	61	11
July	19	55	1.2
August	21	53	13
September	22	53	52
October	24	59	135
November	23	59	240
December	24	67	286
Annual	23	61	1353

Altering the TW or RS application position corresponded to changing application between the two ends of the furrows, which was possible due to the construction of level furrows. It was decided to apply RS followed by complementing TW to the fertigated plots, in which the non-erosive maximum flow rates and flow rates established for this type of system were respected. This method of operation ensured that the RS infiltrated quickly and was only briefly exposed to the soil surface.

**Figure 1:**
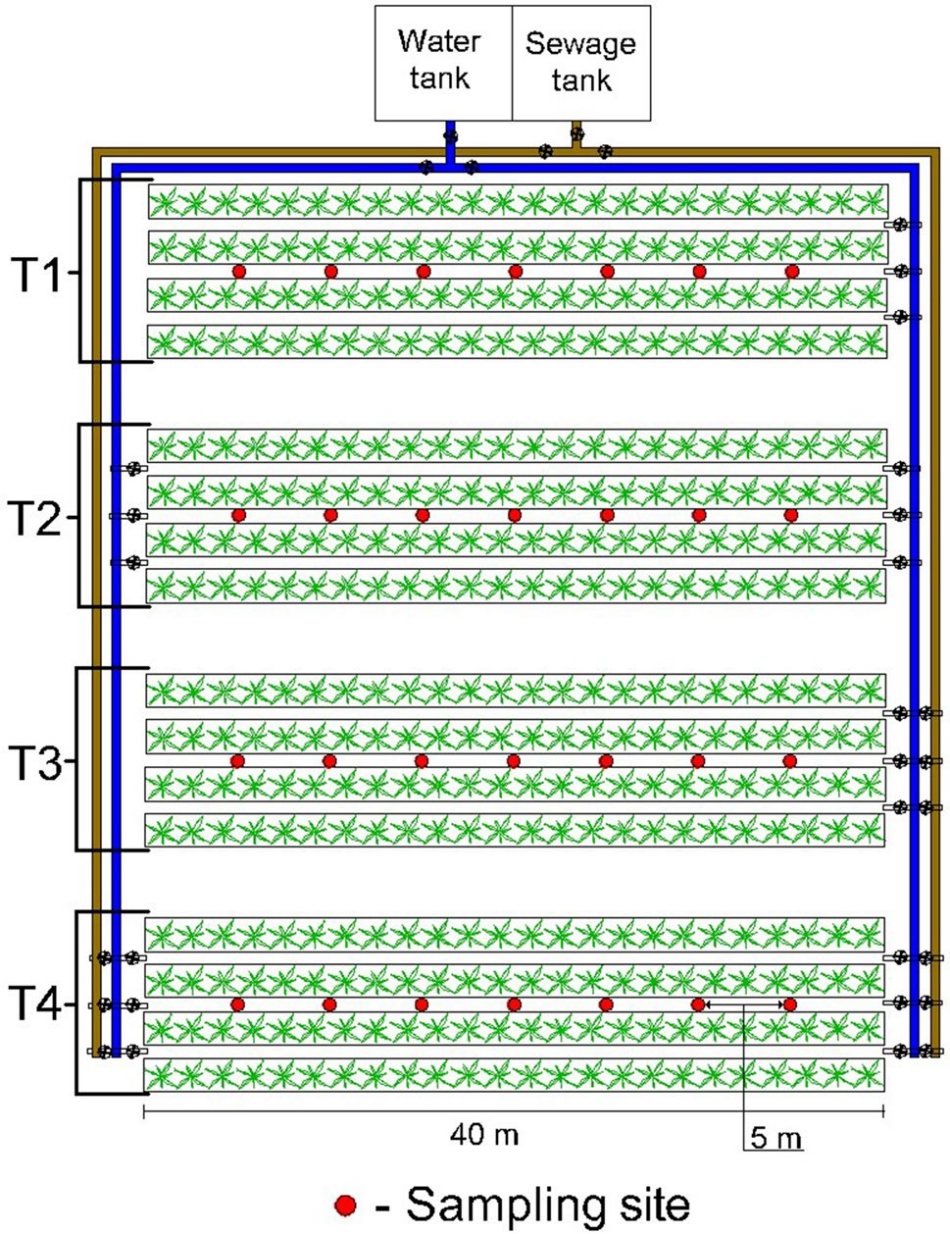
Schematic of the experimental area with indication of the sites where the aerial parts of the plants were sampled.

The experiment was conducted for a period of 3 years, from June 2016 to July 2019 accounted for from the uniform cut of plants, during which periodic cuts of the aerial part of the plants were made whenever the plants of all experimental plots reached a minimum height of 1 m. At each cutting the productivity of the collected material was analyzed in terms of dry matter, where the samples were taken within a 1 m^2^ square, placed every 5 m along the length of the rows. The plants were cut to a minimum height of 15 cm above the ground surface to ensure their regrowth. The collected material was taken to the laboratory and dried at low temperatures (65 °C), and then weighed to obtain the dry matter productivity. Figure 1, presented below, shows the experimental design, indicating the location at which samples of the plant aerial parts were collected.

Samples of the dry aerial part of the plants (stem and leaves), used for productivity calculations, were ground to quantify the total nitrogen (TN), phosphorus (P) and potassium (K) contents, according to the methodology of EMBRAPA^25^. The TN was quantified by the Kjeldahl method after sulfuric digestion; P and K were digested with a nitric-perchloric solution, then P was quantified by colorimetry using visible UV spectrophotometry and K by flame emission spectrophotometry.

The RS was collected weekly before its application to the furrows for fertigation to analyze the sodium (Na), total Kjeldahl nitrogen (TKN), total phosphorus (TP) and total potassium (TK) concentrations. The TKN was quantified by the Kjeldahl method after sulfuric digestion, TP by visible UV spectrophotometry after sulfuric digestion, K by ion chromatography after sulfuric digestion, and Na by ion chromatography after filtering the sample through a 0.22 µm pore diameter filter. All laboratory analyses were performed according to the methodologies proposed by APHA^26^.

Statistical analyses consisted of comparative tests of central tendencies, using the analysis of variance (ANOVA) followed by the Tukey test, in which the level of significance was set at 5%. Comparisons were made between dry matter yields and extracting capacities/average nutrient content in the aerial part of the plants over the experimental period and along the length of the experimental area. Data related to the experimental area was submitted to linear regression analysis, applying the F-test at 5% significance to establish the equation that best predicted the behavior of the analyzed variables. The statistical analyses and graphs presented in this work were generated using the STATISTICA software, version 7.

## Results and discussion

The management adopted in the system guaranteed about 5 cuttings of the plant aerial part per year, which occurred at shorter time intervals between October and March of each year, the period of higher temperatures and greater rainfall intensity, as can be seen in Table 1.^24^

In Table 2 it can be seen that the RS dose equivalent to 300 kg ha^−1^ of Na application provided a N dose about two times that of conventional chemical fertilization. However, the P dose was close to the recommendations, and the K dose was approximately half that suggested by the recommendations of the Minas Gerais State Soil Fertility Commission^21^. Even so, it is considered that the treatments with RS application (TFN and TFA) were sufficient to meet the needs of the plants, where K is a nutrient that potentially limits crop development.

**Table 2:** Mean concentration of sodium (Na) and macronutrients (N, P and K) in raw sanitary sewage, in addition to the amounts applied via fertigation and conventional mineral fertilization in the experimental plots during the 3 year experimental period.

Parameters	Average concentration*	Fertigation	Mineral fertilization
	(mg L^−1^)	Input (kg ha^−1^ year^−1^)	
Na	71.4 (± 21)	300	–
N	148.9 (± 33)	647	300
P	17.8 (± 6)	86	70
K	38.5 (± 11)	176	300

* n = 130; values in parenthesis are the standard deviations.

Considering that the mean sodium concentration in RS was 71.4 mg L^−1^ (Table 2), and the acceptable dose of this chemical element for soil application is 300 kg ha^−1^ year^−1^, at these conditions it was possible to apply approximately 4,202 m^3^ ha^−1^ year^−1^ of this wastewater.

When performing sugarcane fertigation with treated sanitary sewage, reported a contribution of 249 and 33 kg ha^−1^ of N and P, respectively, in one year of application. This difference from the results presented in Table 2 is directly associated with the previous treatment of this wastewater, which tended to significantly reduce the macronutrient concentrations essential for proper plant development^27^.

**Figure 2:**
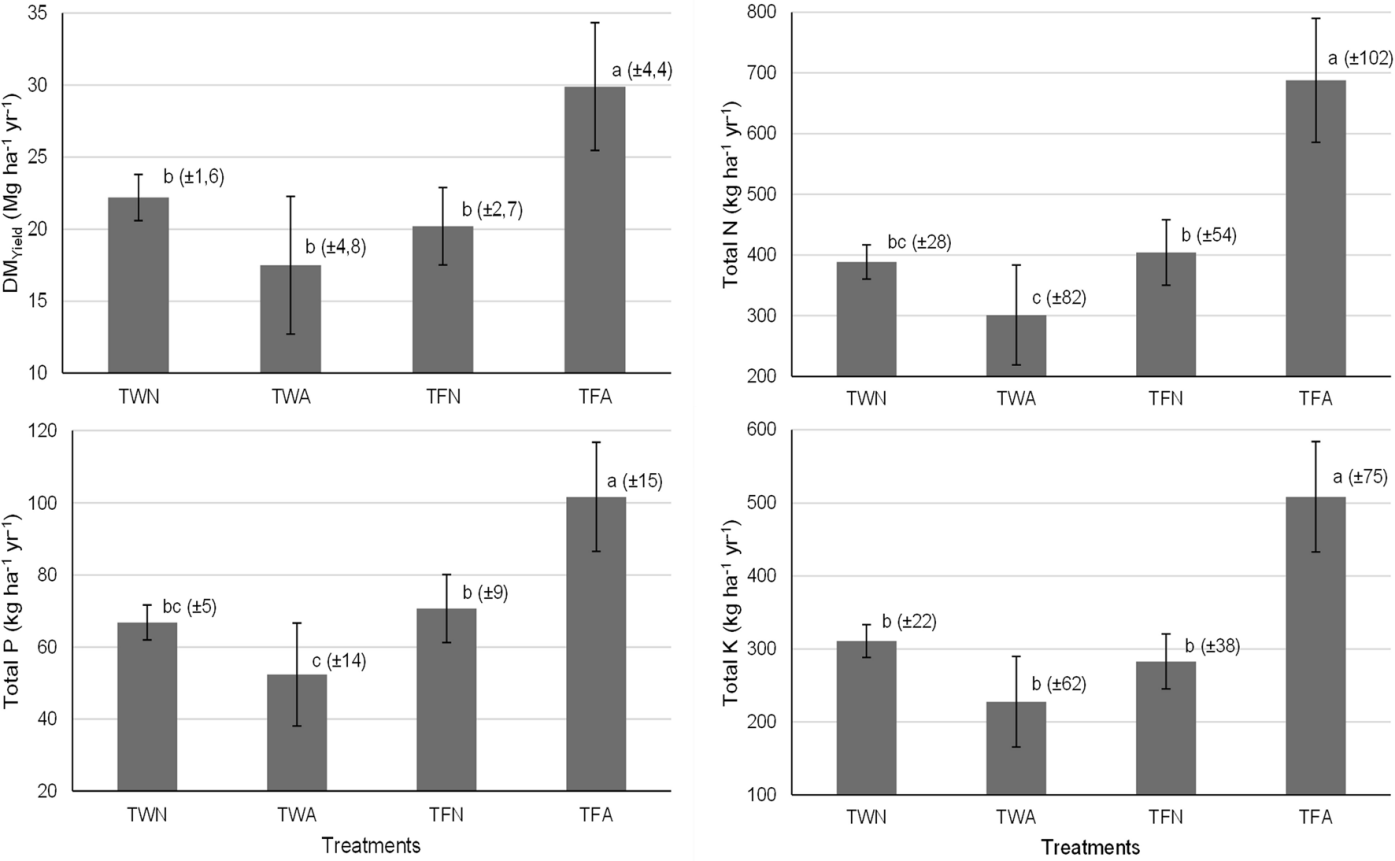
Mean dry matter yield and average macronutrient extraction capacity (extraction of nitrogen, phosphorus and potassium) by the aerial portion of elephant grass over the 3 years of experimentation. Treatments with the same letter have no significant difference according to the Tukey test (p < 0.05, n = 21), while the standard deviations are in parentheses. TWN—Irrigation with water, without alternating the application position in the furrows; TWA—Irrigation with water, with alternation in the application position in the furrows; TFN—Fertigation treatment with RS, without alternation in the application position in the furrows; TFA—Fertigation treatment with RS, with alternation in the application position in the furrows.

The Fig. 2 shows the results of the average dry matter yield of the aerial part of the plants, as well as the respective average amounts of macronutrients allocated in these plant tissues over the 3 years of experimentation. It can be noted that the TFA had the highest dry matter yield, which was 29.9 Mg ha^−1^ year^−1^, indicating that the effect of alternating the RS application position in the closed furrows was positive, increasing productivity by 30% compared to that obtained with the TFN. The treatments TWN, TWA and TFN did not provide significant differences in the dry matter yields of grass aerial parts, with values between 17.5 and 22.2 Mg ha^−1^ year^−1^.

It is believed that productivity of the aerial part of RS-fertigated elephant grass could have been higher if the K needs were met by supplementary chemical fertilization. This is because the crop needs are 300 kg ha^−1^ year^−1^^21^ and, as shown in Table 2, the supply via RS was only 176 kg ha^−1^ year^−1^.

The difference in dry matter yield of elephant grass between the treatments that received CMF and TFA are the result of the difference in N input into the system (Table 2), influencing the rates of macronutrients uptake. Another factor that influenced the better yield in TFA was the form of application of CMF and RS, with CMF being applied only once during the plant cycle, while RS was applied weekly ensuring a better distribution of nutrients.

It was observed that the average amounts of N, P and K extracted were significantly higher in tissue from the aerial part of plants cultivated in the experimental plot submitted to TFA. Values obtained were 688, 102 and 508 kg ha^−1^ year^−1^, respectively, which is due to better distribution of nutrients in the soil resulting from the altering position of RS application in the furrows. The treatments in which the plants received treated water and conventional fertilization (TWN and TWA) were not significantly different and provided maximum uptake N, P and K values of 389, 66.8 and 311 kg ha^−1^ year^−1^, respectively.

In a study conducted in Brazil, it was found that elephant grass was able to extract about 171.8, 55.9 and 1083.6 kg ha^−1^ of N, P and K, respectively, in its aerial portion during a productive period of half a year (189 days). When extrapolating these results for a full year, the authors found similar values of N extraction to those obtained in this experiment for plants submitted to TWN, TWA and TFN treatments, P extraction in the TFA treatment, and higher K values in all treatments. This is explained by the potassium fertilization dose that was used in the cited study^17^.

The macronutrient extraction capacity of the aerial portion of plants submitted to the TFA treatment was higher than the macronutrient dose applied via RS, as can be verified when comparing the results presented in Table 2 and Fig. 2, which guarantees system safety by not allowing nutrients to accumulate in the soil and become susceptible to leaching. The opposite occurred in relation to plants submitted to the TFN, with which an excessive amount of N (647 kg ha^−1^ year^−1^) was applied; these conditions resulted in an average extraction of 404 kg ha^−1^ year^−1^. This indicated that there is a greater risk of poor nutrient distribution in the soil when there is no alternation in the position of RS application to the furrow.

The N extraction capacity of plants submitted to the TFA was 688 kg ha^−1^ year^−1^ (Fig. 2), and the recommendation is 300 kg ha^−1^ year^−1^ via mineral fertilization^21^. This shows that elephant grass has the capacity to extract larger quantities than those recommended by conventional mineral fertilization. According to the study carried out in a tropical climate, doses up to 700 kg ha^−1^ year^−1^ of N result in a linear yield growth of the elephant grass aerial part, presenting an average value of 29 Mg ha^−1^ year^−1^ which is similar to that found in the present study for the aerial part of plants submitted to the TFA^28^.

When evaluating different elephant grass cultivars in different typical Brazilian soils obtained similar results in terms of dry matter yield (about 30 Mg ha^−1^ year^−1^) than those obtained in this work, however nitrogen removal was low (about 157 kg ha^−1^ year^−1^)^29^. Because there was no nitrogen fertilization, the authors associated this N extraction by the aerial part of the plants to biological nitrogen fixation, which could also justify the differences found in relation to this work when considering the quantities applied and extracted by plants submitted to the TFA.

In a study that compared fertigation with treated sanitary sewage and conventional mineral fertilization for the production of bermuda grass, it was found that this wastewater was able to supply up to 30% of the crop nitrogen demands when applied to meet the water demands^30^. The results obtained in this work are indicative of the fact that application of sanitary sewage in its raw form is able to meet the nitrogen needs of elephant grass, considering that it presented a higher N concentration. It can be observed in Fig. 2 that more than 100% of the demand was met, which allowed for greater development of plants submitted to the TFA.

According to the results obtained for macronutrient contents in the aerial part of elephant grass, presented in Table 3, it can be noted that the TFA provided significant differences in N and K contents in relation to those quantified in plants submitted to treatments with application of conventional chemical fertilization (TWN and TWA), and differing only with regards to the K content in the aerial part of plants submitted to the TFN. The P and K contents in the aerial parts are similar to those presented in a study performed with tifton 85 grass, which received fertigation with cattle wastewater at rates of 132 and 402 kg ha^−1^ of P and K, respectively^31^.

**Table 3:** Macronutrient (MN) contents in the aerial portion of elephant grass per unit mass of dry matter (DM).

Treatments	N	P	K
	Percentage per unit mass (%)		
TWN	1.8 b	0.30 a	1.4 b
TWA	1.7 b	0.30 a	1.3 b
TFN	2.0 ab	0.35 a	1.4 b
TFA	2.3 a	0.34 a	1.7 a

Treatments with the same letter have no significant difference according to the Tukey test (p < 0.05, n = 14). TWN—Irrigation with water, without alternating the application position in the furrows; TWA—Irrigation with water, with alternation in the application position in the furrows; TFN—Fertigation treatment with RS, without alternation in the application position in the furrows; TFA—Fertigation treatment with RS, with alternation in the application position in the furrows.

In a study using sanitary sewage submitted to preliminary treatment, P and K contents of up to 0.48 and 3.62% were obtained, respectively, in the dry matter of the aerial portion of coast-cross grass^32^. Corroborating the results obtained in this study, used elephant grass in constructed wetland systems for the treatment of dairy wastewater observed mean levels of 2.81, 0.39 and 1.92% of N, P and K, respectively, in dry matter of the aerial part of the plants^33^.

Using the same Na dose applied to the soil in the present work (300 kg ha^−1^), in fertigation of mombaça grass with preliminary effluent from sanitary sewage treatment, researchers observed levels of N, P and K in the ranges of 1.2—3.5%, 0.9—1.7% and 0.6—2.5%, respectively^14^. Comparing the results presented in Table 3, it was verified that only the P content was below the range found by these authors.

In the treatments where the plants received conventional mineral fertilization, there was less difference in the values of elephant grass dry matter productivity along the length of the experimental areas. This was due to the fact that the chemical fertilizer was applied by spreading, ensuring greater uniformity of its distribution in the area (Table 4).

**Table 4:** Mean dry matter yield and macronutrient extraction by the plants, in function of the length of the area, for the different treatments to which they were submitted during the 3 years of experimentation.

	Lenght (m)							Mean
	5	10	15	20	25	30	35	
Treatments	DM_yield_ (Mg ha^−1^ year^−1^)							
TWN	20.8 bc	21.4 bc	24.2 ab	21.4 b	25.2 a	22.6 b	19.8 b	22.2 b
TWA	17.9 c	17.0 c	18.4 b	18.4 b	16.2 b	16.6 b	17.9 b	17.5 b
TFN	24.2 ab	23.8 ab	23.1 ab	21.3 b	20.3 b	15.0 b	13.7 c	20.2 b
TFA	29.4 a	30.6 a	28.7 a	29.6 a	29.9 a	29.6 a	31.5 a	29.9 a
Treatments	N (kg ha^−1^ year^−1^)							
TWN	334 b	380 b	384 b	409 b	361 b	384 b	444 b	388 bc
TWA	394 b	315 b	349 b	281 c	276 b	172 c	329 c	301 c
TFN	551 a	673 a	471 b	445 b	343 b	250 c	193 d	404 b
TFA	640 a	704 a	703 a	697 a	564 a	811 a	690 a	688 a
Treatments	P (kg ha^−1^ year^−1^)							
TWN	63.5 bc	72.1 c	72.8 c	71.5 c	59.8 b	58.9 b	65.8 b	66.8 bc
TWA	58.7 c	56.4 d	77.7 c	44.0 d	51.7 b	31.0 c	48.0 c	52.3 c
TFN	78.1 a	110 a	89.7 b	89.2 b	53.1 b	51.6 b	34.4 d	70.7 b
TFA	74.8 ab	97.3 b	124 a	110 a	87.5 a	102 a	115 a	102 a
Treatments	K (kg ha^−1^ year^−1^)							
TWN	284 c	264 b	353 b	323 b	386 b	352 b	227 b	311 b
TWA	264 c	240 b	229 c	272 b	117 d	224 c	190 b	227 b
TFN	354 b	319 b	317 b	303 b	298 c	194 c	197 b	283 b
TFA	493 a	541 a	445 a	520 a	501 a	509 a	551 a	508 a

Treatments with the same letter in the column have no significant difference according to the Tukey test (p < 0.05, n = 15). TWN—Irrigation with water, without alternating the application position in the furrows; TWA—Irrigation with water, with alternation in the application position in the furrows; TFN—Fertigation treatment with RS, without alternation in the application position in the furrows; TFA—Fertigation treatment with RS, with alternation in the application position in the furrows.

The same did not occur in the experimental plots in which the plants underwent fertigation with RS without alternation in the application position (TFN), where nutrient extraction by the plants cultivated at the beginning of the furrows was higher which was reflected in the plant development in this area, as shown in Table 4.

The conventional chemical fertilization dose of N and K applied to the experimental plots submitted to TWN and TWA in this experiment was 300 kg ha^−1^ year^−1^, and that of P was 70 kg ha^−1^ year^−1^ (Table 2), which results in extractions of 172 to 444 kg ha^−1^ year^−1^ of N, 31 to 78 kg ha^−1^ year^−1^ of P, and 117 to 386 kg ha^−1^ year^−1^ of K by the plants (Table 4). Thus, the extraction values are consistent with the fertilization recommendations of these nutrients.

The TFN resulted in P extraction by the aerial part of the plants consistent with that applied by RS (86 kg ha^−1^ year^−1^, as shown in Table 2), where the amount extracted was 70.7 kg ha^−1^ year^−1^, although its distribution was not homogeneous along the length of the experimental area. In contrast, the plants submitted to the TFA presented higher P extraction than that applied by RS, indicating that its better distribution along the area made it possible to obtain greater extraction by the plants (Table 4).

Nitrogen, in its nitric form, has become a major concern for furrow irrigation due to its high mobility. Researchers found up to a threefold increase in soil N content when comparing the values quantified at the beginning and end of the experimental area submitted to conventional furrow fertigation (application always at the beginning of furrows), using synthetic wastewater^13^. This difference corroborates what was obtained in the present work in terms of N extraction by plants submitted to TFN (Table 4).

Modeling studies indicate a tendency to reduce the concentration of solutes in water used for fertigation with greater distance from the application point, this is due to their adsorption by the soil^8,9^. Thus, to ensure that the nutritional demand of the plants in the entire area is supplied using RS, it would be necessary to apply a higher dose of this wastewater, contrary to the precepts of environmentally adequate fertigation.

Comparing the extraction capacities of plants submitted to TFN and TFA, it is observed that there began to show significant difference in terms of dry matter productivity at a distance of 20 m from the beginning of the experimental plot area (beginning of the furrows), N extraction capacity at a distance of 15 m, P extraction capacity at a distance of 10 m, and the K extraction capacity over the entire length of this area (Table 4). Thus, it can be affirmed that the differences between the forms of RS application (TFN and TFA) were apparent in the first half of the experimental plot length.

It is important to highlight that agricultural crops have their active root absorption zone in the most superficial soil layer, which prevents obtaining a greater macronutrient extraction capacity when applying RS. Thus, RS application always at the beginning of furrows, as in TFN, results in greater deepening of nutrients and pollutants in the soil profile of this region^7^. As can be seen in Table 4, the plants submitted to TFN were not able to extract the applied N (647 kg ha^−1^ year^−1^) at a distance of 15 m from the RS application site (beginning of the furrows).

According to research, non-absorption of applied N by plants can cause risk of eutrophication of surface and groundwater given the high mobility of this nutrient in the soil^9^. However, studies pointed out that the increase in sinuosity of the furrows, as in the case of their construction following ground level curves, results in less solute loss by deep percolation, an aspect that should be taken into consideration when proposing to use this form of wastewater application to the soil^34^.

Table 5 presents a linear regression analysis of the data provided in Table 4 for the treatments in which RS was applied to the soil (TFN and TFA). It serves to confirm the trends in dry matter productivity and macronutrient extraction capacity by the aerial part of the plants, as well as determination of the best fit equation to explain these performance evaluations as a function of the length of the experimental areas.

**Table 5:** Linear regression analysis of dry matter yield and macronutrient extraction capacity by plants submitted to fertigation with raw sanitary sewage (dependent variables "y") as a function of the distance from the beginning of the furrows in meters (independent variable "x").

Treatments	Adjusted equation	R^2^	P-value	Significance
**DM yield (Mg ha**^−**1**^ **year**^−**1**^)				
TFN	y = − 0.37 x + 27.59	0.8895	0.0014	*
TFA	y = 0.04 x + 29.09	0.2231	0.2845	ns
**Total N (kg ha**^−**1**^ **year**^−**1**^)				
TFN	y = − 14.65 x + 711.01	0.8778	0.0019	*
TFA	y = 1.59 x + 655.24	0.0531	0.6190	ns
**Total P (kg ha**^−**1**^ **year**^−**1**^)				
TFN	y = − 2.04 x + 113.13	0.6746	0.0235	*
TFA	y = 0.66 x + 88.31	0.1815	0.3405	ns
**Total K (kg ha**^−**1**^ **year**^−**1**^)				
TFN	y = − 5.29 x + 388.98	0.8348	0.0040	*
TFA	y = 1.19 x + 484.93	0.1356	0.4164	ns

Statistical analysis by the F-test (p < 0.05; n = 21), where: *—significant regression adjustment; ns—data showed no significant regression adjustment; R^2^—determination coefficient; P-value—probability results of the F-test; TFN—Fertigation treatment with RS, without alternation in the application position in the furrows; TFA—Fertigation treatment with RS, with alternation in the application position in the furrows.

The linear equations adjusted for the TFN presented negative angular coefficient, i.e., they decreased along the furrow length, while those obtained for the TFA presented a positive angular coefficient, but with values close to zero compared to the TFN (Table 5). This behavior corroborates what was previously concluded regarding the better nutrient distribution along the furrows, resulting in higher productivity and higher nutrient extraction capacity of elephant grass submitted to the TFA.

The TFA generated low R^2^ values (less than 30%) compared to the TFN (greater than 80%, except for P) and no significant regression adjustments were obtained (Table 5). The low coefficients of determination of the linear equations indicate low association of dry matter yield and macronutrient removal by the plant aerial portion with the length of the experimental area, i.e., there is a greater balance of nutrient distribution in the area.

Equations adjusted for the TFN explained the phenomena under study, which is the association between reduction in yield and macronutrient extraction by the aerial part of the plant with the length of the experimental area, indicating that when the distance in relation to the RS application site is increased, greater reductions in the contents of the variables under analysis are found. The same behavior was not observed in plants submitted to TFA, indicating that altering the RS application position (TFA) is a good alternative to allow for better plant development in the area.

Since the dry matter productivity, the extraction capacity of all macronutrients studied tended to decrease with the increase in distance from the beginning of the experimental area in plants submitted to the TFN. This reduction in macronutrient extraction by the aerial part of the plant, and consequently plant productivity, is directly associated with poor nutrient distribution in the soil along the experimental plot area, indicated by lower productivity in the end regions of the area^12^.

It is important to note that the productivity and nutrient extraction capacity of plants can be influenced by the flow applied in the furrows and the soil texture. Higher flow rates tend to provide less nutrient precipitation/sedimentation along the length of the furrows, resulting in more uniform distribution in the area. Regarding soil texture, the more clayey the better the nutrient distribution in the area^11^.

## Conclusion

The input of nutrients to the soil with sanitary sewage application was considerable, which makes this wastewater excellent for agricultural use, provided that the fertigation system is properly managed and the recommended application rates are respected.

Successive application of sanitary sewage, with altering its application position at the furrow ends (TFA), provided a significant increase in dry matter yield and macronutrient extraction by the aerial portion of elephant grass, which indicates a better form of managing the furrow fertigation system.

Alternating the position of sanitary sewage application at the different furrow ends provided a 30% increase in the mean dry matter yield when compared to the conventional application method (in a single position at the beginning of the furrows). The values obtained were 20.2 Mg ha^−1^ year^−1^ and 29.9 Mg ha^−1^ year^−1^, respectively, in plants submitted to the non-alternating and alternating treatments (TFN and TFA).

The treatments with alternating position of RS application in the furrows (TFA) provided mean extractions of 688, 102 and 508 kg ha^−1^ year^−1^ of N, P and K, respectively, higher than the amounts extracted by plants submitted to the treatment with no alternation (TFN) and in the conventional chemical fertilizer application treatments (TWA and TWN). This was equal to 100% recovery of the macronutrients added by the application of RS, and therefore the risk of soil leaching was reduced.
